# Effect of segmental muscle vibration on upper extremity functional ability poststroke: A randomized controlled trial

**DOI:** 10.1097/MD.0000000000014444

**Published:** 2019-02-15

**Authors:** Giuseppe Annino, Anas R. Alashram, Alia A. Alghwiri, Cristian Romagnoli, Giuseppe Messina, Virginia Tancredi, Elvira Padua, Nicola Biagio Mercuri

**Affiliations:** a Department of Medicine Systems, University of Rome, “Tor Vergata”; b Department of Human Sciences and Promotion of the Quality of Life, San Raffaele Roma Open University, Rome; c Faculty of Medicine and Surgery, University of Rome, “Tor Vergata,” Italy; d Department of Physical Therapy, School of Rehabilitation Sciences, The University of Jordan, Amman, Jordan; e Alma Mater University, Bologna; f Department of Psychology, Educational Science and Human Movement, University of Palermo, Palermo, Italy.

**Keywords:** function, segmental muscle vibration, stroke, upper extremity

## Abstract

**Background:**

Upper extremity functional impairments are common consequences of stroke. Therefore, continuous investigation of effective interventions for upper extremity functions after stroke is a necessity. Segmental muscle vibration (SMV) is one of the interventions that incorporate sensory stimulation to improve motor cortical excitability. The aim of this study was to investigate the influence of 5-minute SMV application along with supervised physical therapy (SPT) on improving activities of daily living and motor recovery on the hemiparetic upper extremity in patients with stroke.

**Methods:**

A sample of 37 patients poststroke (29 males) was randomly allocated to either SPT control group (n = 18) or SPT and SMV (SPT-SMV) experimental group (n = 19). All patients received 3 sessions per week of SPT for 8 weeks. The SPT-SMV experimental group received SMV at the end of each SPT session. Outcome measures used were Barthel index (BI), modified Ashworth scale, manual muscle testing, and goniometry for range of motion (ROM) assessment.

**Results:**

Thirty-four patients completed the study. Patients in both groups improved significantly after treatment in BI, elbow ROM, and elbow muscles strength. However, muscle tone in elbow joint of the hemiplegic upper extremity improved significantly after SMV only in the experimental group (SPT-SMV).

**Conclusion:**

The SPT intervention can improve functional outcomes of upper extremity in people after stroke. However, using SMV may have superior effect on improving muscle tone after stroke.

## 1 Introduction

Stroke is a leading cause of morbidity and mortality worldwide. According to a recent report, the incidence of stroke across the world ranged from 76 to 119 per 100,000.^[1]^ Around 40% of patients poststroke exhibit moderate functional impairments while 15% to 30% exhibit severe disabilities.^[2]^ While a majority of those who suffer from a stroke regain independent mobility, the hemiplegic arm only regains motor function and return of task-specific activities of daily living (ADL) <15% of the time.^[3]^ About 80% of stroke survivors require upper extremity therapy^[4]^ with 40% of survivors experience moderate to severe deficits in the upper extremity.^[5]^

Common manifestations of upper extremity motor impairment include muscular weakness, contracture, and tone changes as well as impairments in motor control.^[6]^ Almost every patient who experiences a cerebral or brainstem stroke develops a physical disability that affects ADL, including eating, dressing, and personal hygiene.^[7]^ Grip strength, another important variable needed for ADL, is also typically affected.^[8]^ Limitations in ADL greatly reduce independence, social participation, and quality of life.^[9]^ As time progresses patients can regain some motor function originally lost. It was thought that dynamic recovery only occurred up to 6 months poststroke; however, new therapies are illustrating that motor recovery can continue after that.^[10–12]^ Studies have shown that in order for rehabilitation to be effective, therapy needs to be highly repetitive, promote afferent input, and be functional, as well as engage the user and encourage frequent practice.

Therapeutic strengthening exercises and task-oriented training are among the common methods used to correct or prevent musculoskeletal deficiencies and improve the function and ADL through neuromuscular adaptations.^[13]^ The American Heart and Stroke Association recommends that strength training be conducted at 50% to 80% of the 1-repetition maximum for 10 to 15 repetitions for 2 to 3 days per week and that resistance be increased as tolerance permits for people with stroke.^[14]^

Segmental muscle vibration (SMV) is a fairly new technique that has been used to improve motor function^[15]^ and inhibit spasticity in the hemiplegic upper extremity of patients following a stroke.^[16]^ In SMV, a vibratory stimulus is applied to a specific muscle tendon using a mechanical device unit in which it induces the generation of Ia inputs as a consequence of the activation of muscle spindle primary endings.^[17]^ Vibration of a muscle can increase the motor-evoked potential recorded from the muscle at rest,^[18]^ suggesting enhancement of corticospinal excitability changes during vibration.^[19,20]^ An increased duration of cortical silent period in a forearm flexor muscle during vibration of the antagonist forearm extensors has been exhibited^[21]^ and evidence strongly suggests that a period of pure sensory stimulation can affect motor cortical excitability.^[22]^

Considering the effect of vibration stimulus on the neuromuscular system, the aim of this study was to investigate the influence of a 5-minute SMV application along with a supervised physical therapy (SPT) protocol on ADL and motor recovery (range of motion [ROM], muscle tone, and muscle power) of elbow joint on the hemiparetic side in patients with stroke.

## 2 Methods

### 2.1 Participants

The study was designed as a single randomized controlled trial for a convenience sample of 37 patients postischemic stroke (7 females, 30 males) was selected based on the study inclusion and exclusion criteria. An inclusion criterion was a confirmed diagnosis of stroke. Patients were excluded from this study if they had any serious orthopedic conditions not related to stroke, cardiopulmonary problems or suffered from a neurological disease (other than stroke).

Written informed consent was obtained from participants before they were randomly assigned to either SPT control group (n = 18) or SPT-SMV experimental group (n = 19). The study was approved by University of Rome Tor Vergata ethics committee with a protocol number (212/17) and was registered in clinicalTrials.gov with an ID (NCT03419793).

### 2.2 Procedures

Participants were recruited from private rehabilitation center (Tlaa al ali center, Amman, Jordan). Fifty-two patients were contacted medical examination. Thirty-seven met the inclusion criteria and allocated to 2 different groups using an automated computer randomization program. Two patients in SPT-SMV group dropped out of the intervention due to personal circumstances. One patient in SPT control group dropped out due to inability to participate in exercise training. Thirty-four patients were completed the study.

Baseline evaluation and posttreatment measures were performed by 1 clinical examiner blinded to the intervention. Demographic and health-related information were collected from each participant including age, gender, weight, height, duration of stroke, family history of stroke, type of stroke, and hemiplegic side.

All participants received 3 sessions per week of SPT for 8 weeks in private rehabilitation center. Each session of SPT protocol consisted of 30 minutes of manual resistance exercise (3 sets/10 repetitions each for elbow extensors and flexors) and functional training for the hemiplegic upper extremity. The SPT-SMV experimental group received the segmental muscle vibrator (SMV) at the end of each SPT session. The SMV was applied for 5 minutes perpendicularly along the muscle fibers of the triceps muscle with frequency of 30 Hz and amplitude of 2 mm.

### 2.3 Outcome measures

All participants were assessed at baseline and reassessed at the end of week 8 of treatment. The main outcome was Barthel index (BI). Secondary outcomes were modified Ashworth scale (MAS), manual muscle testing (MMT), and goniometry for ROM assessment.

The MAS was utilized to measure spasticity.^[23]^ The scale asses the resistance of limb to a rapid passive stretch in 6 scores from 0 to 5. Score 0 indicates normal muscle tone, and 5 indicates rigid limb. We tested flexion and extension of elbow joint on hemiplegic side.

The goniometer tool was used to measure active ROM (AROM).^[24]^ The goniometer axis were secures on the lateral epicondyle for measuring elbow ROM. The patient was 1st asked to actively move the elbow to full extension and then full flexion. Patient was asked to maintain full extension and flexion for 3 seconds each and the median angles of the two 3-second intervals were used for AROM calculation.

The MMT was used to assess muscle strength.^[25]^ The MMT consists of 6 grades.^[26]^ Grade 0 indicates no evidence of contractility (complete paralysis), and 5 indicates movement against gravity plus full resistance. We tested flexors and extensors for elbow on hemiplegic side.

The BI was used to measure performance in ADL.^[27]^ The BI consists of 10 variables; bowels, bladder, help needed with grooming, toilet use, feeding, transfers, walking, dressing, climbing stairs, and bathing. The total scores range from 0 to 100, with lower scores indicating increased disability.^[28]^

### 2.4 Statistical analysis

The nonparametric test for 2 independent samples (Mann–Whitney *U* test) was used to examine the mean difference in outcome measures between the SPT-SMV experimental group and the SPT control group at the end of treatment. The nonparametric test for 2 dependent samples (Wilcoxon-signed ranks test) was used to examine the mean difference in outcome measures within each group (SPT-SMV and SPT groups) in the period between baseline and end of treatment. Effect sizes were calculated to identify the difference between pre- and posttest values within group using the following formula: *r* = *Z*/√N (*Z*: *Z*-value, N: number of observations), where small effect was 0.1, moderate was 0.3, and large was 0.5.^[29]^ Statistical analysis was conducted using SPSS statistics version 20.

## 3 Results

**Figure 1:**
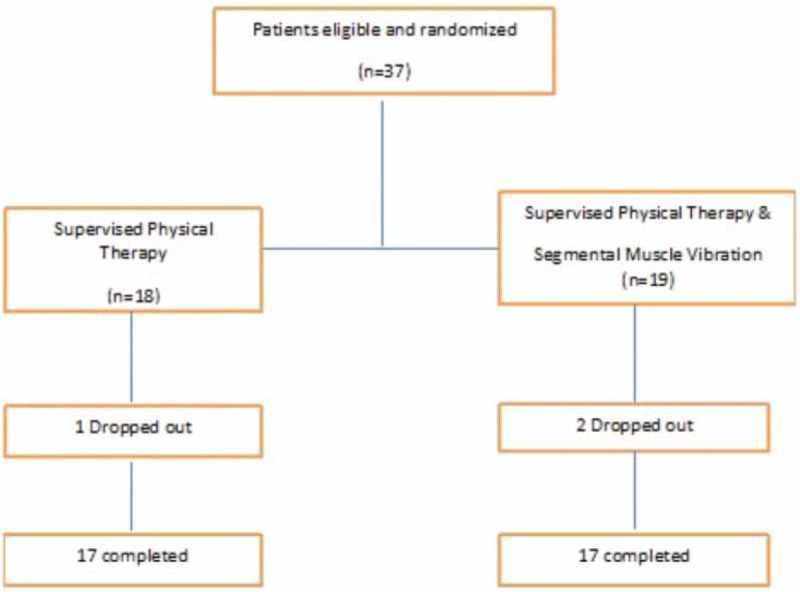
Flow of participant through the trial.

During the 8-week program, 2 participants from the SPT-SMV group and 1 participant from SPT group dropped out of the study. Reasons for dropping out were due to personal circumstances and being unable to participate in exercise training. Thirty-four participants (17 in SPT-SMV group and 17 in SPT group) completed the study (Fig. 1). Demographic and health-related characteristics of participants are presented in Table 1. No significant differences were found between the 2 groups neither in demographic information nor in outcome measures at baseline.

**Table 1:**
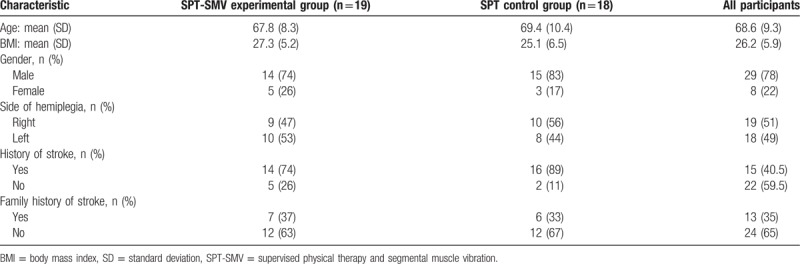
Characteristics of participants (N = 37).

Table 2 presents the means and standard deviations of outcome measure scores in the treatment (SPT-SMV) and control (SPT) groups during baseline and at the end of treatment. Participants in both groups improved significantly after treatment in BI, elbow ROM, and elbow muscles strength. However, muscle tone in elbow joint of the hemiplegic upper extremity improved significantly after SMV only in the experimental group (SPT-SMV). After investigating the differences between groups at the end of treatment, there were no significant differences found in any of the outcome measures.

**Table 2:**
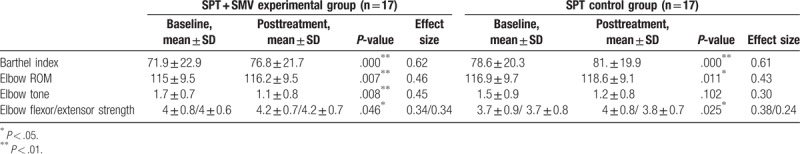
Mean and standard deviation (SD) of outcome measures for supervised physical therapy and segmental muscle vibration (SPT-SMV) experimental group and supervised physical therapy (SPT) control group at baseline and posttreatment (N = 34).

## 4 Discussion

In this study, we investigated the effect of segmental body vibrator and physical therapy interventions on the hemiplegic upper extremity in stroke patients. Similar to the results of our study, Caliandro et al found that applying vibration on the spastic limb of chronic stroke participants produced a difference in motor function when tested using the Wolf motor function test (2012).^[15]^ SMV has been shown to activate muscle spindles and thereby create Ia afferent fiber firing when it is used on a tendon-muscle component.^[17]^ This firing of Ia fibers changes the corticospinal pathway's excitability^[30]^ and activates the motor areas of the cortex.^[31]^ Celletti et al surmised that by utilizing a multidisciplinary approach of vibration therapy and rehabilitation techniques with a neurophysiologic base, a rebalancing can occur within the cortical excitatory and inhibitory system (2017).^[32]^

In 2012, Noma et al demonstrated significant improvement in *F*-waves and MAS immediately following as well as 30 minutes following application of SMV.^[16]^ Our findings are consistent with several studies that revealed that SMV, especially in combination with physiotherapy, had a significant impact on muscle tone in patients with chronic stroke; frequently these changes are long lasting and had a significant impact on muscle tone with a carryover into motor function.^[33]^

Our study showed that when using SMV in combination with physical therapy interventions, muscle tone, mainly, improved more significantly than with exercises alone. This was also noted by Tavernese et al, who reported that the use of SMV on the biceps brachii and flexor carpi ulnaris of the affected side of their participants who had suffered a stroke, along with physical therapy, produced a significant improvement in normalized jerk and reaching motion and this effect was maintained at the participants’ 2-week posttreatment evaluation.^[34]^ In the present study, the improvement in muscle tone has contributed to a noted improvement in ADL independence.

In our study, we assessed the movement of “reaching” for 2 main reasons: reaching is the upper extremity movement that creates the most interaction between the hand and the surrounding environment^[28]^ and because of the extent that this motion has been studied in participants who have had a stroke. Typically, the movement of reaching for patients following a stroke is characterized by an increase in movement duration, a reduction in the velocity of execution of the movement as well as a decrease in the smoothness of the movement.^[35–37]^ A combined treatment of SMV and SPT has been shown to produce a significant improvement in motor performance in the paretic upper limb during reaching movement in a population of patients with chronic stroke.^[34]^

### 4.1 Limitations

The results of present study should be evaluated taking into account some limitations that should be acknowledged. First, our present study is that it is not a double-blinded study. However, using of an instrumented measure for outcomes assessment and blinded of independent assessor to the groups partially limited this bias. Secondly, present RCT included a small number of patients. Future studies should have a larger sample size to confirm our results. Lastly, the patients affected by chronic stroke were tested only before and after the 8-week treatment protocol without a long follow-up. To understand the effect high-frequency vibrations in poststroke patients, future studies should include a long follow-up.

## 5 Conclusion

Our data suggest that a multidisciplinary treatment approach to chronic ischemic stroke patients by combining segmental muscle vibrator with physical therapy interventions show significant reduction of spasticity and determine significant improvement in ADLs. In this context SMV appears to be a useful device in adjunct to physical therapy on the treatment of spasticity in stroke patients. However, further studies are needed to verify this hypothesis, specifically increasing the time of exposure to vibration treatment per session, as well as increasing the number of participants.

## Author contributions

**Formal analysis:** Alia A. Alghwiri, Virginia Tancredi.

**Investigation:** Giuseppe Annino.

**Methodology:** Giuseppe Annino.

**Resources:** Cristian Romagnoli.

**Software:** Giuseppe Messina.

**Supervision:** Nicola Biagio Mercuri.

**Visualization:** Elvira Padua.

**Writing – original draft:** Anas R. Alashram.

Giuseppe Annino orcid: 0000-0001-8578-6046.

## References

[1] Thrift AG Thayabaranathan T Howard G Global stroke statistics. International journal of stroke: official journal of the International Stroke Society 2017;12:13–32. 27794138 10.1177/1747493016676285

[2] Duncan PW Zorowitz R Bates B Management of adult stroke rehabilitation care: a clinical practice guideline. Stroke 2005;36:e100–43. 16120836 10.1161/01.STR.0000180861.54180.FF

[3] Simkins M Kim H Abrams G Robotic unilateral and bilateral upper-limb movement training for stroke survivors afflicted by chronic hemiparesis. IEEE Int Conf Rehabil Robot 2013;2013:6650506. 24187321 10.1109/ICORR.2013.6650506

[4] Friedman N Chan V Reinkensmeyer AN Retraining and assessing hand movement after stroke using the MusicGlove: comparison with conventional hand therapy and isometric grip training. J Neuroeng Rehabil 2014;11:76. 24885076 10.1186/1743-0003-11-76 PMC4022276

[5] Paquin K Crawley J Harris JE Survivors of chronic stroke - participant evaluations of commercial gaming for rehabilitation. Disabil Rehabil 2016;38:2144–52. 26728133 10.3109/09638288.2015.1114155

[6] Hatem SM Saussez G Della Faille M Rehabilitation of motor function after stroke: a multiple systematic review focused on techniques to stimulate upper extremity recovery. Front Hum Neurosci 2016;10:442. 27679565 10.3389/fnhum.2016.00442 PMC5020059

[7] Anderson TP Stolov WC Clowers MH Stroke and cerebral trauma: medical aspects. Diane Publishing Co, Handbook of Severe Disability. Washington, DC: 1981.

[8] Sunderland A Tinson D Bradley L Arm function after stroke. An evaluation of grip strength as a measure of recovery and a prognostic indicator. J Neurol Neurosurg Psychiatry 1989;52:1267–72. 2592969 10.1136/jnnp.52.11.1267 PMC1031635

[9] Nudo RJ Plautz EJ Frost SB Role of adaptive plasticity in recovery of function after damage to motor cortex. Muscle Nerve 2001;24:1000–19. 11439375 10.1002/mus.1104

[10] Page SJ Levine P Back from the brink: electromyography-triggered stimulation combined with modified constraint-induced movement therapy in chronic stroke. Arch Phys Med Rehabil 2006;87:27–31. 16401434 10.1016/j.apmr.2005.07.307

[11] Page SJ Levine P Modified constraint-induced therapy extension: using remote technologies to improve function. Arch Phys Med Rehabil 2007;88:922–7. 17601475 10.1016/j.apmr.2007.03.038

[12] Page SJ Levine P Modified constraint-induced therapy in patients with chronic stroke exhibiting minimal movement ability in the affected arm. Phys Ther 2007;87:872–8. 17472950 10.2522/ptj.20060202

[13] Guanabara Koogan, Hall CM Brod LT Exercícios terapêuticos na busca da função, 2nd ed. 2007.

[14] Billinger SA Arena R Bernhardt J Physical activity and exercise recommendations for stroke survivors: a statement for healthcare professionals from the American Heart Association/American Stroke Association. Stroke 2014;45:2532–53. 24846875 10.1161/STR.0000000000000022

[15] Caliandro P Celletti C Padua L Focal muscle vibration in the treatment of upper limb spasticity: a pilot randomized controlled trial in patients with chronic stroke. Arch Phys Med Rehabil 2012;93:1656–61. 22507444 10.1016/j.apmr.2012.04.002

[16] Noma T Matsumoto S Shimodozono M Anti-spastic effects of the direct application of vibratory stimuli to the spastic muscles of hemiplegic limbs in post-stroke patients: a proof-of-principle study. J Rehabil Med 2012;44:325–30. 22402727 10.2340/16501977-0946

[17] Roll JP Vedel JP Ribot E Alteration of proprioceptive messages induced by tendon vibration in man: a microneurographic study. Exp Brain Res 1989;76:213–22. 2753103 10.1007/BF00253639

[18] Mileva KN Bowtell JL Kossev AR Effects of low-frequency whole-body vibration on motor-evoked potentials in healthy men. Exp Physiol 2009;94:103–16. 18658234 10.1113/expphysiol.2008.042689

[19] Rosenkranz K Rothwell JC Differences between the effects of three plasticity inducing protocols on the organization of the human motor cortex. Eur J Neurosci 2006;23:822–9. 16487162 10.1111/j.1460-9568.2006.04605.x

[20] Smith L Brouwer B Effectiveness of muscle vibration in modulating corticospinal excitability. J Rehabil Res Dev 2005;42:787–94. 16680616 10.1682/jrrd.2005.02.0041

[21] Binder C Kaya AE Liepert J Vibration prolongs the cortical silent period in an antagonistic muscle. Muscle Nerve 2009;39:776–80. 19334048 10.1002/mus.21240

[22] Ford B Fahn S Pullman SL Peripherally induced EMG silent periods. Normal physiology and disorders of motor control. Adv Neurol 1995;67:321–8. 8848978

[23] Bohannon R Smith M Interrater reliability of a modified Ashworth scale of muscle spasticity. Phys Ther 1987;67:206–7. 3809245 10.1093/ptj/67.2.206

[24] Gajdosik R Bohannon R Clinical measurement of range of motion. Phys Ther 1987;67:1867–72. 3685114 10.1093/ptj/67.12.1867

[25] Fan E Ciesla N Truong A Inter-rater reliability of manual muscle strength testing in ICU survivors and simulated patients. Intensive Care Med 2010;36:1038–43. 20213068 10.1007/s00134-010-1796-6 PMC2891143

[26] Angel M Bril V Shannon P Neuromuscular function in survivors of the acute respiratory distress syndrome. Can J Neurol Sci 2007;34:427–32. 18062450 10.1017/s0317167100007307

[27] O'Sullivan SB Schmitz TJ Physical Rehabilitation, 5th ed. 2007;Philadelphia, PA: FA Davis Company, 385.

[28] Mahoney F Barthel D Functional evaluation: the Barthel index. Md Med J 1965;14:61–5. 14258950

[29] Rosenthal R Cooper H Hedges LV Valentine JC Parmetric measures of effect size. The Handbook of Rsearch Syenthesis. New York: Russell Sage Foundation; 1994 231–44.

[30] Steyvers M Levin O Van Baelen M Corticospinal excitability changes following prolonged muscle tendon vibration. Neuroreport 2003;14:1901–5. 14561917 10.1097/01.wnr.0000093296.63079.fa

[31] Fourment A Chennevelle JM Belhaj-Saif A Responses of motor cortical cells to short trains of vibration. Exp Brain Res 1996;111:208–14. 8891651 10.1007/BF00227298

[32] Celletti C Sinibaldi E Pierelli F Focal muscle vibration and progressive modular rebalancing with neurokinetic facilitations in post-stroke recovery of upper limb. Clin Ter 2017;168:e33–6. 28240760 10.7417/CT.2017.1979

[33] Marconi B Filippi GM Koch G Long-term effects on cortical excitability and motor recovery induced by repeated muscle vibration in chronic stroke patients. Neurorehabil Neural Repair 2011;25:48–60. 20834043 10.1177/1545968310376757

[34] Tavernese E Paoloni M Mangone M Segmental muscle vibration improves reaching movement in patients with chronic stroke. A randomized controlled trial. NeuroRehabilitation 2013;32:591–9. 23648613 10.3233/NRE-130881

[35] Wu C Trombly CA Lin K A kinematic study of contextual effects on reaching performance in persons with and without stroke: influences of object availability. Arch Phys Med Rehabil 2000;81:95–101. 10638883 10.1016/s0003-9993(00)90228-4

[36] Caimmi M Carda S Giovanzana C Using kinematic analysis to evaluate constraint-induced movement therapy in chronic stroke patients. Neurorehabil Neural Repair 2008;22:31–9. 17595381 10.1177/1545968307302923

[37] Liebermann DG Levin MF McIntyre J Arm path fragmentation and spatiotemporal features of hand reaching in healthy subjects and stroke patients. Conf Proc IEEE Eng Med Biol Soc 2010;2010:5242–5. 21096047 10.1109/IEMBS.2010.5626297

